# Falls caused by balance disorders in the elderly with multiple systems involved: Pathogenic mechanisms and treatment strategies

**DOI:** 10.3389/fneur.2023.1128092

**Published:** 2023-02-23

**Authors:** Liwei Xing, Yi Bao, Binyang Wang, Mingqin Shi, Yuanyuan Wei, Xiaoyi Huang, Youwu Dai, Hongling Shi, Xuesong Gai, Qiu Luo, Yong Yin, Dongdong Qin

**Affiliations:** ^1^School of Basic Medical Sciences, Yunnan University of Chinese Medicine, Kunming Yunnan, China; ^2^The First Clinical Medical School, Yunnan University of Chinese Medicine, Kunming Yunnan, China; ^3^Department of Rehabilitation Medicine, The Affiliated Hospital of Yunnan University, Kunming Yunnan, China; ^4^Department of Rehabilitation Medicine, The Third People's Hospital of Yunnan Province, Kunming Yunnan, China; ^5^Department of Rehabilitation Medicine, The First People's Hospital of Yunnan Province, Kunming Yunnan, China

**Keywords:** balance, fall, elderly, pathogenesis, treatments, mechanism

## Abstract

Falls are the main contributor to both fatal and nonfatal injuries in elderly individuals as well as significant sources of morbidity and mortality, which are mostly induced by impaired balance control. The ability to keep balance is a remarkably complex process that allows for rapid and precise changes to prevent falls with multiple systems involved, such as musculoskeletal system, the central nervous system and sensory system. However, the exact pathogenesis of falls caused by balance disorders in the elderly has eluded researchers to date. In consideration of aging phenomenon aggravation and fall risks in the elderly, there is an urgent need to explore the pathogenesis and treatments of falls caused by balance disorders in the elderly. The present review discusses the epidemiology of falls in the elderly, potential pathogenic mechanisms underlying multiple systems involved in falls caused by balance disorders, including musculoskeletal system, the central nervous system and sensory system. Meanwhile, some common treatment strategies, such as physical exercise, new equipment based on artificial intelligence, pharmacologic treatments and fall prevention education are also reviewed. To fully understand the pathogenesis and treatment of falls caused by balance disorders, a need remains for future large-scale multi-center randomized controlled trials and in-depth mechanism studies.

## Introduction

Falls are the leading cause of injury-related mortality among the elderly globally, trailing only traffic accidents in prominence (1), and increasing the mortality rate and disability rate in the elderly. Approximately 27,000 older adults died due to falls in a year (2). Elderly fall victims may endure severe physical harm, including fractures, especially under the circumstance of prior hip surgery or osteoporosis. They may also experience loss of independence and are forced into nursing home admittance (3). Thus, fearfulness of falling might cause social withdrawal and disengagement (4). Psychological and physical injuries resulting from falls impose a heavy social and financial strain on patients' family, community health services, and economy. Consequently, preventing falls in the elderly is a pressing public health issue.

One of the leading causes of falls in the elderly is balance disorders, which frequently result in harm, disabling conditions, loss of independence, and lowered quality of life (5). With several systems cooperating together to prevent falls, good balance is likely the result of a quick synergistic interplay between diverse physiologic and cognitive factors that enables a speedy and precise reaction to the perturbation (6). Elderly people are more inclined to fall due to balance disorders resulting from the steady reduction of several systems' functions, including musculoskeletal system, the central nervous system and sensory system (7–10). The present review discusses the epidemiology of falls in the elderly, potential pathogenic mechanisms underlying multiple systems involved in falls caused by balance disorders, including musculoskeletal system, the central nervous system and sensory system, as well as some common treatment strategies for balance-disorder-induced falls by regulating different systems.

## Epidemiology

Approximately 28 to 35% of individuals over 65 years of age fall each year, and 32–42% of individuals over 70 years of age fall, according to the WHO's (world health organization) survey. This demonstrates that the risk of falling increases with age (11). 20–30% of mild–severe injuries are the results of falls (12), and more than 50% of such injuries require hospitalization for treatment (13). Among them, about 35% of individuals over 70 years of age and 61% of individuals over 80 years of age have suffered from balance disorders (14). With increasing age, greater mobility difficulty, decreased cognitive function, living alone, more concomitant conditions, and the likelihood of experiencing multiple falls increased dramatically (15). A cross-sectional study on the prevalence and risk factors for falls among the elderly indicates that the associated factors of falls among older adults includes impaired balance ability, less physical activity, cognition impairment, mild and moderate depression (16). In conclusion, falls are common in the elderly, which is the result of a complicated pathological process involving a multitude of factors.

## Pathogenic factors with multiple systems involved

**Figure 1:**
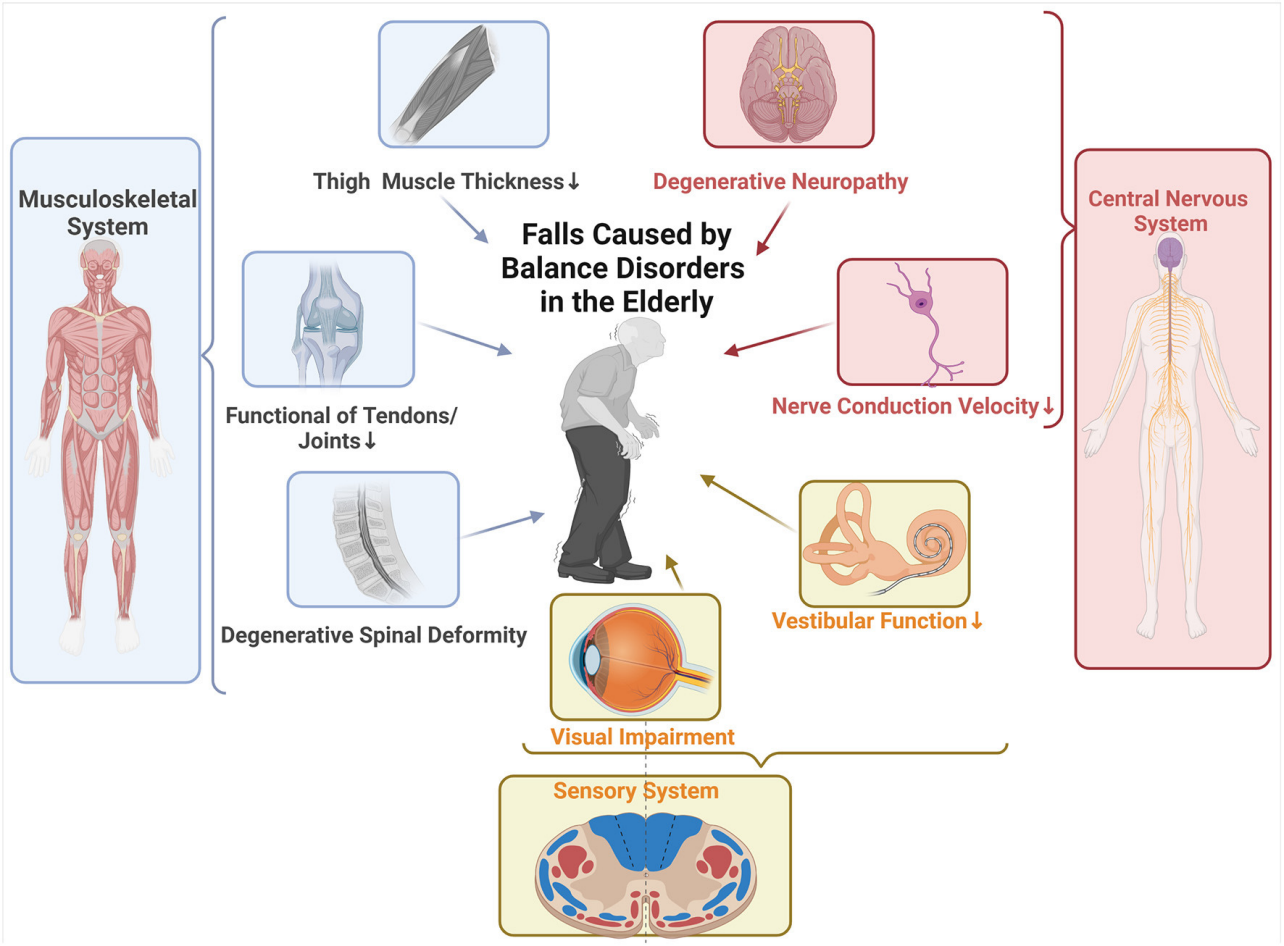
Summary of pathogenic factors with multiple systems involved in falls caused by balance disorders in the elderly. Musculoskeletal system: In the elderly, decreased thigh and core muscle thickness is favorably connected with dynamic balance and correlated with fall risk. Functional degradation of tendons and joints occurs with aging, which is characterized by decreased muscle mass and reduced contractility, and thus leads to falls. Degenerative spinal deformity, which develops in the elderly due to accumulated degenerative changes brought on by aging, such as asymmetrical disc degeneration, dehydration, and collapse, combined with facet degeneration and ligamentous laxity, can significantly alter the body's center of gravity and cause falls. Central nervous system (CNS): Aging brings about the spinal networks' structural changes that impair their functionality and motor commanding. Unmyelinated fiber density decreases by 37% in the elderly, while myelinated fiber density decreases by 38%, which is the main culprit of elderly's diminished nerve conduction velocity. Physical balance disorders and an elevated risk of falling are caused by the CNS's degeneration, which makes it more difficult for the body to integrate motor signals. Sensory System: The visual system and vestibular system are like GPS and sentry mod. Older people with visual impairment have a higher incidence of falls than those without visual impairment. The decline in vestibular function with increasing age has been confirmed by many studies and thus can be classified as one of the causes of falls.

Balance is the ability to maintain the projected center of mass of the body within the stability limits of support, which has three fundamental properties, such as steadiness, symmetry, and dynamic stability (17). In addition to the aging of physiological functions, balance disorders in the elderly also represents the aggregation of pathology in multiple systems, all of which can result in falls (18). Identifying the pathogenic factors of falls caused by balance disorders in the elderly are the premise and basis for the identification, assessment, and control of dysfunction and loss of independence, which are shown briefly in Figure 1.

### Musculoskeletal system

From a biomechanical point of view, when a body is stationary on a plane, its center of mass and pressure are in a vertical projection that touches the support surface (8). While, when the upright balance is lost, with the shifting center on the wrong supporting surface, falls happen (8, 19). The coordination of skeletal muscles throughout the body helps to maintain the forementioned balance dynamically and statically by impacting biomechanical parameters such as body sway, stride length, stride frequency and symmetry (20–23). Thigh and core muscle thickness is favorably connected with dynamic balance and negatively correlated with fall risks (24). However, functional degradation of tendons and joints occurs with aging, which is characterized by decreased muscle mass and reduced contractility, thus leading to falls (25). Elderly people alter the joint torque of their ankle, knee, and hip joints, as well as their body balance, *via* coronal and sagittal adjustments (26). Degenerative spinal deformity, which develops in the elderly due to accumulated degenerative changes, such as asymmetrical disc degeneration, dehydration, and collapse, combined with facet degeneration and ligamentous laxity, can significantly alter the body's center of gravity and cause falls (27).

A study indicates that balance disorders in the severe stage of myasthenia are considerably greater than those in non-myasthenia and pre-myopathy, and the chance of falling is higher, demonstrating a strong correlation between the severity of myasthenia and the risk of balance disturbance (28). Sarcopenia, or the loss of muscle mass with aging, is mostly caused by a decrease in the size of the fast muscle fibers (type II), which results in a higher proportion of slow muscle fibers (type I) in elderly patients (18). Therefore, in older people, the muscles' ability to generate force and their contractile characteristics are more uniform. Aged muscles do, in fact, exhibit a decreased functional working range and are still unable to contract quickly. A study indicates that falls are associated with high levels of muscle activation, which are traits of age-related losses in postural stability (29).

The intake of vitamin D also plays an indispensable role in the balance disorders induced by skeletal muscle. Conventionally, vitamin D plays a major role in regulating calcium and phosphorus metabolism. When vitamin D is deficient, bone density and hardness are reduced, making fractures and falls more likely to happen (30). For the link between muscle and vitamin D, many people have focused on the vitamin D receptor (VDR) although VDR cannot be detected in skeletal muscle (31). However, recent studies have shown that muscles can be regulated by VDR, including atrophy, regeneration, and repairment (32–34). Most studies have shown that vitamin D can predict skeletal muscle health in senior adults and improve lower limb muscle mass and function (35–37). However, it is still unclear how vitamin D functions in muscle, even though it has been documented that muscle function improves after supplementation of vitamin D.

### The central nervous system

Besides the muscular and skeletal control in falls, the presence of the central nervous system (CNS) is often not negligible. When the CNS including afferent and efferent pathways are damaged, such as stroke, spinal cord injury or long-term bedridden, significant changes in the muscle mass will cause myasthenia and eventually lead to falls (38–40). At the same time, the spinal networks' structural changes that impair their functionality and motor command are also brought on by aging. Unmyelinated fiber density decreases by 37% in the elderly, while myelinated fiber density decreases by 38% (41). This is the main culprit of elderly's diminished nerve conduction velocity (42). Physical balance disorders and an elevated risk of falling are caused by the CNS's degeneration, which makes it more difficult for the body to integrate motor signals.

The anterior tibialis muscle, a dorsal foot flexor, is thought to have more efferent pathway degradation than other foot flexors in the elderly, but isometric muscular strength does not appear to be affected until 80 years of age. This is possible because the muscles are collaterally re-innervated by intact nerve endings, which expands the size of the functional remaining motor units (43). The maximum isometric torque (MIT) of the ankle muscles correlates adversely with center of foot pressure (CoP) displacements (43). It is considered that neuropathy-related injury in proprioceptive sensation of ankle and excessive burden of torque development rate in the elderly lead to the decrease of balance function, and thus eventually cause falls (44).

### Sensory system

It is now widely known that aging affects the sensory systems involved in the body's orientation and stabilization in space (45). The visual system and vestibular system are like GPS (global positioning system) and sentry mod. As the “GPS” of the human body, the visual system can let us know whether there is danger around us through the input of environmental information, ensure our safe movement, and also predict and give feedback in different directions and spaces (10). A survey of older people in communities found that older people with visual impairment had a higher incidence of falls than those without visual impairment (46). There is also a large proportion of vision loss due to other causes besides aging. Glaucoma, cataract and other diseases that lead to vision loss plague the elderly, which also leads to an increase in falls (47, 48). As the “sentry mod” of the body, the vestibular system is responsible for standing, movement, balance and control of navigation (10). The vestibular system consists of three parts, namely the homogeneous and bony labyrinths, the motion sensors of the vestibular system, and the hair cells (49). The cooperation of extraocular and vestibular functions through the vestibulo-ocular reflex (VOR) helps the body compensate the head and stabilize the image (49, 50). In vestibular-loss patients, when the eyes are closed, it is difficult for the human body to compensate and they are more inclined to fall (51). The decline in vestibular function with increasing age has been confirmed by many studies and thus can be classified as one of the causes of falls (52, 53).

The proprioception and tactility can be complementary to vestibular perception and vision and can also serve as another source of information to the CNS to more accurately control the balance of the body (10, 54). Firstly, muscles, tendons and joints all provide different kinds of information for us to keep our balance and prevent from falling. Muscle spindle is the primary kinesthetic sensor (55). A study of patients with hereditary sensory and autonomic neuropathies (HSANs) found that the absence of functional muscle spindles afferents caused ataxia (56). In addition, the sense of touch is also a subtle but essential part of maintaining balance. Balance scores are also decreased in patients with reduced plantar sensation after stroke, which can be corrected *via* weight transfer between the legs, especially with the eyes closed (57). In patients with diabetes, researchers have found a significant relationship between sensory loss and falls due to peripheral nerve loss in the feet (58). One study of older adults over 60 years old showed that the risk of recurrent falls in patients with loss of sensation was 3.59 times than that of controls (59).

Recent research has shown that multisensory integration plays an important role in balance management as well. In general, the maintenance of balance does not depend on the above parts alone. Balance maintenance should be viewed as a collaborative process involving multiple systems, with different weights assigned to different components in different tasks (60). Multisensory integration is the process through which the nervous system combines data from several perceiving processes, including hearing, feeling, and other somatosensory events, into a single, unified, coherent, and stable multisensory process (61). Studies have shown that, with aging, the multisensory integration is progressively impaired and probably results in falls (62). Insufficient multisensory re-weighting, which is crucial for postural control in senior persons, has been linked to poor balance control in elderly people who are prone to falling (25, 63). A clinical study determining the association of multisensory integration with mobility outcomes in aging indicates that magnitude of multisensory integration is an incremental predictor of incident fall, over and above balance and other known fall risk factors (64). The prefrontal cortico-cortico facilitation, dedifferentiation, and prefronto-thalamo-cortical gatin have been linked to decreased information processing in an aged brain, which may be the cause of this condition (65). Collectively, balance maintenance should be viewed as a collaborative process involving multiple systems.

## Treatments

As previously mentioned, falls caused by balance disorders in the elderly are complicated and involve multiple systems. Thus, targeted considerations must be taken when treating and rehabilitating elderly patients with postural balance issues. Treatments of balance-disorder-induced falls by regulating different systems were summarized in this review and succinctly outlined in Table 1 (66–76).

**Table 1:** Summary of studies indicating treatment strategies of balance-disorder-induced falls by regulating different systems in the elderly.

**Type**	**Treatment strategies**	**Corresponding systems**	**Assessment indicators**	**Results**	**Reference**
Physical exercise	Monochromatic infrared energy (MIRE) exposure and Tai Chi exercise	Musculoskeletal system	Berg balance scale (BBS), tinetti clinical scale (TCS), timed up and go test (TUG)	Statistically significant improvements in balance and reduction in the risk of falls in community-dwelling older adults	(61)
	Mobility, strength, coordination, and balance exercise	Musculoskeletal system, central nervous system, and sensory system	Tinetti test and short physical performance battery (SPPB)	Statistically significant improvements in balance and reduction in the risk of falls	(62)
	Chinese fitness dancing	Musculoskeletal system	Maximum muscle strength, fall risk index, and static balance ability of extensor muscle groups in the lower limbs	Statistically significant improvements in muscle strength in the lower limbs and effectively lowered the fall risks	(63)
New equipment based on artificial intelligence	New techniques for retraining based on the feedback technology	Musculoskeletal system, central nervous system, and sensory system	Miniexamen cognoscitivo test, oddball test, attention network test, timed up and go test	Statistically improvements in balance, gait, autonomy, and fall risk	(64)
	Virtual reality (VR) program and motor imagery training (MIT)	Musculoskeletal system, central nervous system, and sensory system	Body center movement area, open and closed eyes balance scores, and fall efficacy	Significant improvements in body center movement area, open and closed eyes balance scores, and fall efficacy	(65)
	Center-of-pressure (COP) controller	Musculoskeletal system, central nervous system	Gait stability and electromyography for muscle activity	Statistically significant improvements in gait stability	(66)
Pharmacologic treatments	Vitamin D	Musculoskeletal system	Berg balance test and biodex balance system (postural stability and fall risk tests)	Statistically significant improvements in balance	(67)
	Antiparkinsonian medication	Central nervous system, and sensory system	12-month incidence rate ratio (IRR) of falls	Antiepileptics were associated with falls [IRR 2.16 (95% CI 1.10-4.24)]	(68)
Fall prevention education	Exercise training combined with education	Musculoskeletal system, central nervous system, and sensory system	Fall efficacy, physical activity, and lower extremity muscle strength	Statistically significant improvements in fall efficacy, physical activity, and lower extremity muscle strength	(69)
	Educational intervention	Musculoskeletal system, central nervous system, and sensory system	Thai Fall Risk Assessment Tool (Thai-FRAT)	Statistically significant reduction in balance impairment, medicine usage, and falls' overall incidence	(70)
	Education and exercise	Musculoskeletal system	Falls efficacy, muscular strength	Significantly fewer falls, less stiffness, less difficulty performing activity; more muscular strength, walking ability, and balance	(71)

### Physical exercise

The most effective method to decrease the rate of falls, enhance gait ability, keep balance, and strengthen performance in physically fragile older persons appears to be a multi-component exercise intervention that combines strength, endurance, and balance training (77). Multi-system physical exercise (MPE) is composed of four parts, including proprioceptive training, muscle strength training, reaction training and postural balance training, which can help to restore the function of musculoskeletal system, the CNS and sensory system. A study has shown that under MPE intervention training even without poorly supervised balance and endurance training (78), elderly people over 65 years old who are at risk of falling show significant improvement in all four aspects and muscle strength is significantly increased, and the fall risk reduced (79). At the same time, Tai Chi (TC) is also helpful for improving balance and can prevent falls in the elderly due to requiring more muscle strength of the lower limb joints. Therefore, the body can develop neuromuscular control strategies to maintain body balance and thus reduce the risk of falls (80). Also intense physical activity boosts levels of brain-derived neurotrophic factor (BDNF), slows down the loss of brain tissue, increases hippocampal capacity, boosts cerebral blood flow, and enhances CNS performance, including executive functions, restoring balance and lowering the risk of accidents (81).

### New equipment based on artificial intelligence

New equipment based on artificial intelligence have been introduced to enrich the whole recovering process. Robot assisted training (RAGT) can bring many benefits to patients, including muscle strength, power, range of motion and so on. Patients with poststroke ankle spasms could significantly improve ankle spasms and increase balance after RAGT intervention (82). Virtual reality can be combined with RAGT to improve patients' gait (83). This combination enhances the function of musculoskeletal system, the CNS and sensory system in the elderly. Recently, a large number of fall detection system (FDS) have been developed, which can be divided into the following three categories: video-based (84), ambient sensor-based (85) and wearable sensor-based (86). A study invented the class-imbalanced deep learning fall detection (CDL-Fall) with a specificity of 91.86%, an F-Score of 98.44%, which is effective on class-imbalanced data and more suitable for real-life application algorithm (87). It has been demonstrated that a novel form of shoe insert called SoleSensor^®^ (U.S. patent issued in 2001, licensed to Hart Mobility, Inc.), can improve the ability of sensory system in the elderly, which is effective in preventing falls (88). In developing novel, cost-effective interventions aimed at identifying specific balancing systems in the elderly, greater attention needs to be paid to target and implement artificial intelligence.

### Pharmacologic treatments

Beyond improved bone health, vitamin D helps to prevent falls and fractures. Strengthening muscles with vitamin D helps lowering the risk of falling. According to a meta-analysis, supplementing with vitamin D at a dose of 700 to 1000 IU per day lowers the risk of falling in older people by 19% (89). Before beginning supplementing, the doctor should also find out whether the elderly patient is using any over-the-counter medications that include vitamin D, as too much vitamin D might cause hypercalcemia. The practical strategy is to promote a vitamin D-rich, healthy, balanced diet (90). In the elderly, polypharmacy is quite common, and thus a lot of side effects from clinically drug-drug interaction emerges, including orthostatic hypotension, dizziness, and somnolence, all of which can lead to falls. According to a study, there was a 39% decrease in the rate of falling when psychotropic medicines, such as benzodiazepines, other sleep aids, neuroleptic agents, and antidepressants, were tapered and stopped over the course of a 14-week period (91). Besides, balance disorder induced by neurally mediated hypotension should get specialized treatments and prescriptions (92).

### Fall prevention education

The education of fall prevention is a necessary part of the whole strategy and runs through the whole process. A meta-analysis of six fall prevention education (FPE) programs indicated that FPE intervention reduced the incidence of fall-related behaviors among community-dwelling residents (93). The content of the education generally includes: the definition of falls, the prevalence of falls, risk factors, and complications of falls. The final goal is to make patients aware of their current situation and how to adjust and change by themselves, and seek the help of care-giver (93). Especially for the elderly with fear of falling (FOF), the combination of FPE and other therapies has greatly reduced their FOF, thus reducing the occurrence of falls (94).

## Summary and outlook

Multiple systems, including the musculoskeletal system, the CNS and sensory system, are all involved in the mechanism underlying the increased occurrence of falls caused by balance disorders in the elderly. Contemporary research, however, has mostly focused on the parallels and correlations between the various systems, rather than the basic processes of falls brought on by balance impairments in the elderly. Few randomized controlled trials and animal model experiments, in contrast, thoroughly examine the mechanism of the efficient treatment approach. Previous research has shown that physical exercise, new technology based on artificial intelligence, pharmacological treatments, and fall prevention education can effectively treat falls caused by balance disorders in the elderly. However, due to varied approaches and a lack of randomized controlled studies with a high sample size, there is currently a lack of useful data supporting the use of these strategies to particularly target certain systems. Future large-scale multicenter randomized controlled trials, in-depth mechanistic research, including big multicenter trials are still required to completely understand the underlying mechanism and management of falls brought on by balance disorders in the elderly.

## Author contributions

All authors listed have made a substantial, direct, and intellectual contribution to the work, and approved it for publication.
